# Exploring the Mechanism of Indigo Naturalis in the Treatment of Ulcerative Colitis Based on TLR4/MyD88/NF-κB Signaling Pathway and Gut Microbiota

**DOI:** 10.3389/fphar.2021.674416

**Published:** 2021-07-22

**Authors:** Qi-yue Yang, Le-le Ma, Chen Zhang, Jun-zhi Lin, Li Han, Ya-nan He, Chun-guang Xie

**Affiliations:** ^1^ TCM Regulating Metabolic Diseases Key Laboratory of Sichuan Province, Hospital of Chengdu University of Traditional Chinese Medicine, Chengdu, China; ^2^ School of Pharmacy, Chengdu University of Traditional Chinese Medicine, Chengdu, China

**Keywords:** indigo naturalis, indigo, indirubin, ulcerative colitis, gut microbiota

## Abstract

**Background:** Clinical trials have proven that indigo naturalis is a candidate drug for treating ulcerative colitis (UC), but its therapeutic mechanism is still unclear.

**Purpose:** This study aimed to evaluate the protective effect and mechanism of indigo naturalis to treat mice with dextran sulfate sodium (DSS)-induced UC.

**Methods:** DSS-induced UC mice were treated with indigo naturalis (200 mg/kg), indigo (4.76 mg/kg), and indirubin (0.78 mg/kg) for 1 week. The anti-UC mechanism of indigo naturalis was studied by pathological section, inflammatory factor, western blot, and 16S rRNA sequencing.

**Results:** According to body weight change, disease activity index, and colon length, indigo naturalis had the strongest anti DSS-induced UC effect, followed by indirubin and indigo. Pathological section showed that indigo naturalis, indigo, and indirubin could reduce the infiltration of inflammatory cells, increase the secretion of intestinal mucus, and repair the intestinal mucosa. Indigo naturalis, indigo, and indirubin could reduce IL-1β,IL-6, and TNF-α by inhibiting TLR4/MyD88/NF-κB signal transduction. Indigo naturalis and indigo could also reduce IgA and IgG both in serum and colon tissue. In addition, indigo naturalis, indigo, and indirubin could adjust the gut microbiota structure of DSS-induced UC mice, reducing the ratio of Firmicutes/Bacteroidetes and increasing the abundance of probiotics.

**Conclusion:** Indigo and indirubin are one of the main anti-UC components of indigo naturalis. INN could regulate intestinal flora, reduce inflammation, repair intestinal mucosa, and improve the physiological status of DSS-induced UC mice and its anti-UC mechanism may be involved in inhibiting TLR4/MyD88/NF-κB signal transduction.

## Introduction

Ulcerative colitis (UC) is a chronic and nonspecific intestinal inflammatory disease with unknown etiology, characterized by recurrent abdominal pain, diarrhea, and bloody stool (Gajendran et al., 2019). The incidence rate of UC is 0.03–0.1% and is increasing every year (Harbord et al., 2017). At present, the treatment of UC mainly depends on 5-aminosalicylic acid, steroids, glucocorticoids, immunosuppressants, and biological agents (Kornbluth and Sachar, 2010; Ford et al., 2011; Rogler, 2013). However, the dependence, drug resistance, intolerance, loss of reaction, and opportunistic infection of these drugs are increasingly becoming new clinical problems (Renna et al., 2014). Simultaneously, their remission rate is not satisfactory in clinical trials. Therefore, complementary and alternative therapy has been an important issue for UC patients, clinicians, and researchers.

Herbal medicine is the prime choice of complementary and alternative therapy for UC. In recent years, Japan has been actively exploring single herbs for treating UC. They found that indigo naturalis, a traditional Chinese medicine extracted from natural blue plants, can effectively treat refractory cases of intestinal mucosal aplasia. Then follow-up high evidence-based clinical research was carried out and indigo naturalis was confirmed to be effective for all kinds of UC and demonstrated a good application prospect (Torisu et al., 2019; Matsuno and Hirano, 2020; Naganuma et al., 2020; Yoshimatsu et al., 2020). However, the mechanism of indigo naturalis is not clear, which may be related to the inhibition of nitric oxide release, the prevention of superoxide production, the repair of intestinal mucosa, and the regulation of intestinal flora (Saito et al., 2015; Liang and Yu, 2019; Ozawa et al., 2020; Yoshimatsu et al., 2020). Exploring the mechanism of indigo naturalis in the treatment of UC is helpful to apply correctly in clinic and reduce the side effects in the process of medication.

The pathogenesis of UC is related to the immune system. Toll-like receptors (TLRs) are one of the pattern recognition receptors in the innate immune system; its imbalance of regulation can aggravate mucosal injury in acute inflammation (Zhou et al., 2017). TLR4 is an important member of TLRs family. Intestinal innate immune response initiated by external stimulation can up-regulate the expression of TLR4 and activate NF-κB through MyD88 dependent signaling pathway, resulting in severe abnormalities of intestinal mucosal epithelium (Cheng et al., 2018; Gu et al., 2018). It is common to find that TLR4/MyD88/NF-κB signaling pathway is abnormally activated in the intestinal mucosa of UC patients (Ke et al., 2013). In a previous study, indigo and indirubin in idigo naturalis was shown to have higher affinity for TLR4, MyD88, and NF-κB receptors than other receptors through molecular docking, especially MyD88 (Supplementary Material 1). Therefore, this study explored the effects of indigo naturalis in UC by TLR4/MyD88/NF-κB signaling pathway. Additionally, the interaction of microorganisms *in vivo* determines the health of the body, especially in the colon where microorganisms are abundant (Franzosa et al., 2019). Studies have shown that the intestinal mucosal inflammatory response caused by various factors is accompanied by the change of intestinal microecology and the imbalance of flora (Kostic et al., 2014). In this article, we further discussed the effect of indigo naturalis on intestinal flora, in order to provide more scientific support for indigo naturalis in the treatment of UC.

## Materials and Methods

### Materials and Reagents

Indigo naturalis (INN) was purchased from Sichaun Jiangyou Hengyuan Pharmaceutical Co., Ltd (Jiangyou, China). It was determined by HPLC that INN contains 2.38% indigo (IND) and 0.39% indirubin (INB). Standards of IND and INB (≥98%, purity) were purchased from Chroma-Biotechnology Co., Ltd (Chengdu, China). The enzyme-linked immunosorbent assay (ELISA) kits for TNF-α, IL-1β, IL-6, IgA, IgG, IgM, and myeloperoxidase (MPO) were purchased from MultiScience (Lianke) Biotech Co., Ltd. Dextran sulfate sodium (DSS, 36–50 KDa) was purchased from MP Biomedicals (Aurora, United States). Sulfasalazine (SSZ) was purchased from Shanxi Tongda pharmaceutical Co., Ltd (Shanxi, China). Rabbit anti-TLR4 antibody, anti-TLR2 antibody, anti-NF-κB p65 antibody, anti-NF-κB p-p65, anti- IKBα antibody, anti-p-IKBα antibody, anti-β-actin antibody, MyD88 antibody, anti-Histone H3 antibody, and other antibodies were purchased from Servicebio Biotechnology Co., Ltd (Wuhan, China).

### Animals

Male BalB/c mice (20 ± 2 g) were purchased from Chengdu Dashuo Laboratory Animal Co., Ltd (Permit No. SCXK (chuan) 2015-30, Chengdu, China). The animals were kept under controlled conditions of temperature 20 ± 0.5˚C, humidity 55 ± 5%, and a 12 h light/dark cycle.

### Therapeutic Potential of INN, IND, and INB on a DSS-Induced UC Mouse Model

The mice were randomly divided into six groups: Normal group, DSS group, INN group, IND group, INB group, and SSZ group (*n* = 6). After 1 week of adaptive feeding, the mice in DSS, INN, IND, INB, and SSZ groups were given 3% DSS drinking water (w/v) to establish UC model and mice in the normal group were given distilled water. After drinking DSS solution for 3 days, mice in INN, IND, INB, and SZZ groups were given intragastric administration of INN (200 mg/kg), IND (4.76 mg/kg), INB (0.78 mg/kg), and SSZ (200 mg/kg) respectively. All mice received DSS drinking water for 7 days and drug therapy for 7 days. The weight and feces of mice were monitored and recorded every day throughout the experiment. Further, the health status of mice was evaluated according to the disease activity index (DAI), which is normal stool (0), soft stools (1), soft stools and slight bleeding (2), loose stools and slight bleeding (3), and gross bleeding (4).

After 10 days of administration, mice in all groups were taken eyeballs to collect blood. Serum was isolated from blood for ELISA. Then, all mice were sacrificed and their colon and feces samples were collected. The colon length of each mouse was measured and divided into three parts for pathological analysis, ELISA, and western blot analysis. The preparation of hematoxylin and eosin (H&E) and periodic acid-Schiff (PAS) pathological sections was commissioned by Servicebio Biotechnology Co., Ltd (Wuhan, China). Pathological changes of colonic tissue were observed under microscope, and the degree of colitis was evaluated according to the standard of Table 1. The tissue damage index (TDI) was calculated as TDI = (score of inflammatory cell infiltration + score of inflammatory cell infiltration depth + score of ulcer depth)/3. The levels of IL-1, IL-6, TNF-α, IgA, IgG, IgM, and MPO in serum and colon were analyzed by ELISA according to the manufacturer’s instructions.

**TABLE 1:** TDI score standard

Inflammatory cell infiltration	Inflammatory cell infiltration depth	Ulcer depth	Score
/	/	/	0
Mild	Mucosa	Epithelium	1
Medium	Mucosa and submucosa	Lamina propria mucosa	2
Severe	Whole colon	Muscularis mucosae	3

### Western Blot Analysis

The colon tissues were lysed for 30 min in RIPA buffer (Servicebio, Wuhan, China) with protease and phosphatase inhibitors on ice. The lysates were centrifuged at 12,000 rpm for 10 min at 4°C. The precipitate was removed and the supernatant was obtained as total protein. According to the instructions, the concentration of total protein was determined by BCA Protein Assay Kit (Servicebio, Wuhan, China). The protein from each sample was separated using 10% SDS-PAGE electrophoresis and transferred on nitrocellulose membrane (Millipore Corporation, United States) at 25 V overnight. The membrane was blocked in 5% non-fat milk in TBST (0.5%) for 1 h at room temperature and incubated with appropriate rabbit anti-TLR4 antibody, anti-TLR2 antibody, anti-NF-κB p65 antibody, anti-NF-κB p-p65, anti- IKBα antibody, anti-p-IKBα antibody, anti-β-actin antibody, and anti-Histone H3 antibody at a 1:1,000 dilution, and anti-MyD88 antibody at a 1:2,000 dilution overnight at 4°C. The membrane was washed with TBST three times, 5 min each time. Then, it was incubated with HRP-labeled secondary antibody, which was detected by chemiluminescence substrate.

### The Effect of INN, IND, and INB on Gut Microbiota

The total genomic DNA in mice feces was extracted by DNeasy PowerSoil Kit (QIAGEN, Germany), and the genomic DNA was detected by 1% agarose gel electrophoresis. The V3-V4 variable region sequence of the bacterial 16S rRNA gene was used as the target, and 338F-806R with barcode sequence as a primer was used to amplify the PCR products. The PCR products were recovered and purified by AxyPrep DNA Gel Extraction Kit (Axygen Biosciences, United States), and then washed by Tris-HCl and detected by 2% agarose gel electrophoresis. According to preliminary quantitative results of electrophoresis, QuantiFluor™-ST (Promega, United States) was used for quantitative analysis. The purified amplicons were mixed in equal amount, and the double terminal sequencing was carried out according to the standard process of Illumina PE250 sequencing platform.

The original data is submitted to NCBI sequence read Archive (SRA) database. The sequencing fragment was clustered into operational taxonomic units (OTU) by QIIME 2.0 software based on a 97% sequence similarity. OTU representative sequences were compared with Silva database to obtain the corresponding taxa (including phylum, genus, and species) and their abundance information. Then, QIIME, Mothur, R software, and Python LEfSe package were used for OTU data to analyze the alpha and beta diversity, species composition, and difference, which could provide a reference for further exploring the function of flora and analyzing the mechanism of action.

### Statistical Analysis

All data were expressed as mean ± SD and analyzed by SPSS 21.0 statistical software. If the data were in accordance with normal distribution and the variance was homogeneous, one-way ANOVA was used; otherwise, rank sum test was used. *p* < 0.05 and *p* < 0.01 were considered statistically significant.

## Results

### The Effect of INN, IND, and INB on DSS-Induced UC in Mice

**FIGURE 1:**
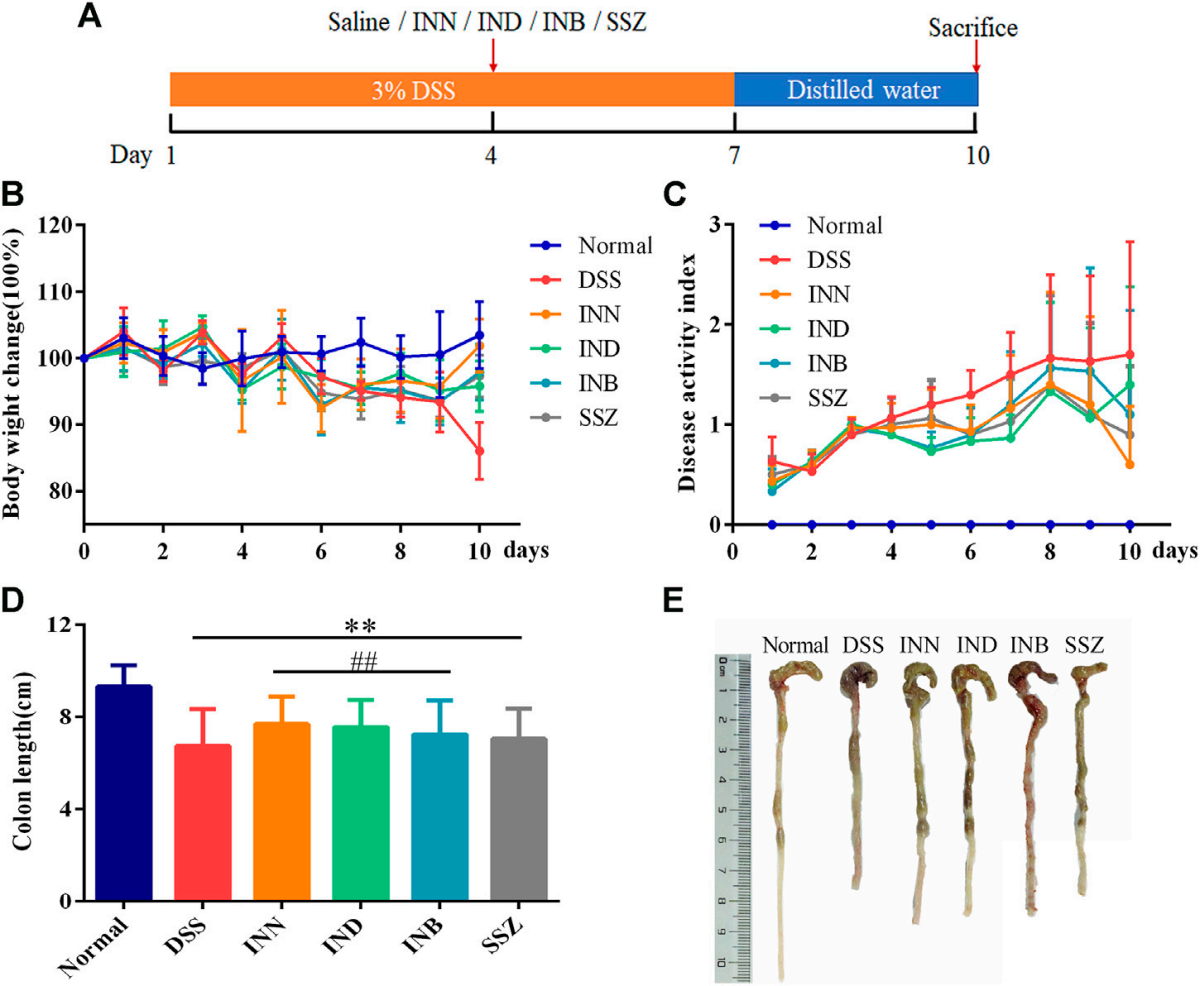
Effects of INN, IND, INB, and SZZ treatment on the of DSS-induced UC mice after 7 days of continuous gavage. **(A)** The medication regimen; **(B)** body weight change in various groups; **(C)** disease activity index in various groups; **(D)** colon length in various groups; **(E)** photos of colon length in various groups. *vs* normal group, **p < 0.01; *vs* DSS group, ^*##*^ *p* < 0.01. Data are expressed as mean ± SD (*n* = 6).

After drinking 3% DSS solution for 3 days, mice showed obvious UC symptoms, namely diarrhea, bloody stool, and other symptoms. After drinking 3% DSS solution for 7 days, the health of mice was very bad without drug intervention. Therefore, drug intervention was started on the fourth day after drinking 3% DSS solution (Figure 1A). Changes in weight of mice reflected their health status. Compared with normal mice, weight was lost significantly in DSS-induced mice (Figure 1B). Mice treated with INN, INB, IND, and SSZ showed less weight loss compared with the DSS group. DAI evaluation showed that the DAI score in DSS-induced mice was obvious raised (Figure 1C). After treating with INN, IND, INB, and SSZ, the DAI score significantly decreased, indicating that these drugs could improve UC status, especially INN. Furthermore, the colon length was obviously shortened in DSS-induced mice (Figures 1D,E)). INN, IND, and INB can prevent the colon shortening.

### The Histopathological Changes in Colon Tissues

**FIGURE 2:**
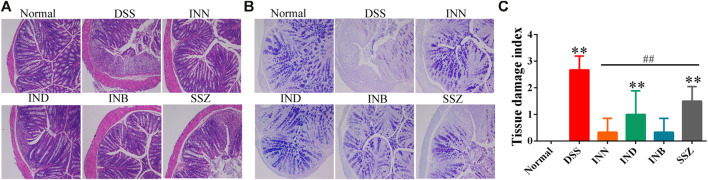
Histological sections of colonic tissue stained with HE **(A)** and PAS **(B)** and TDI score **(C)** (magnification ⅹ 100).

In the normal group, the colon section had a normal mucus secretion without necrosis and inflammation (Figure 2). The colon of mice in the DSS group had obvious ulcers, in which a mucosal layer, intestinal glands and epithelium were completely lost, accompanied by a large number of inflammatory cells infiltrating into the submucosa and muscular layer (Figure 2A). After treating with INN, IND, and INB, the inflammatory cell infiltration was reduced and the integrity of intestinal mucosal epithelial cells was improved, especially in INN and INB groups (Figure 2C). PAS staining showed that expressions of mucins in DSS group were lower, illustrating that intestinal mucosa was damaged and the secretion of intestinal mucus was insufficient (Figure 2B). After administration of INN, IND, INB, and SSZ, mucin expressions were raised. These results indicated that INN, IND, and INB could repair intestinal mucosa and increase intestinal mucus secretion.

### The Changes of Inflammatory Cytokine Both in Serum and Tissue

**FIGURE 3:**
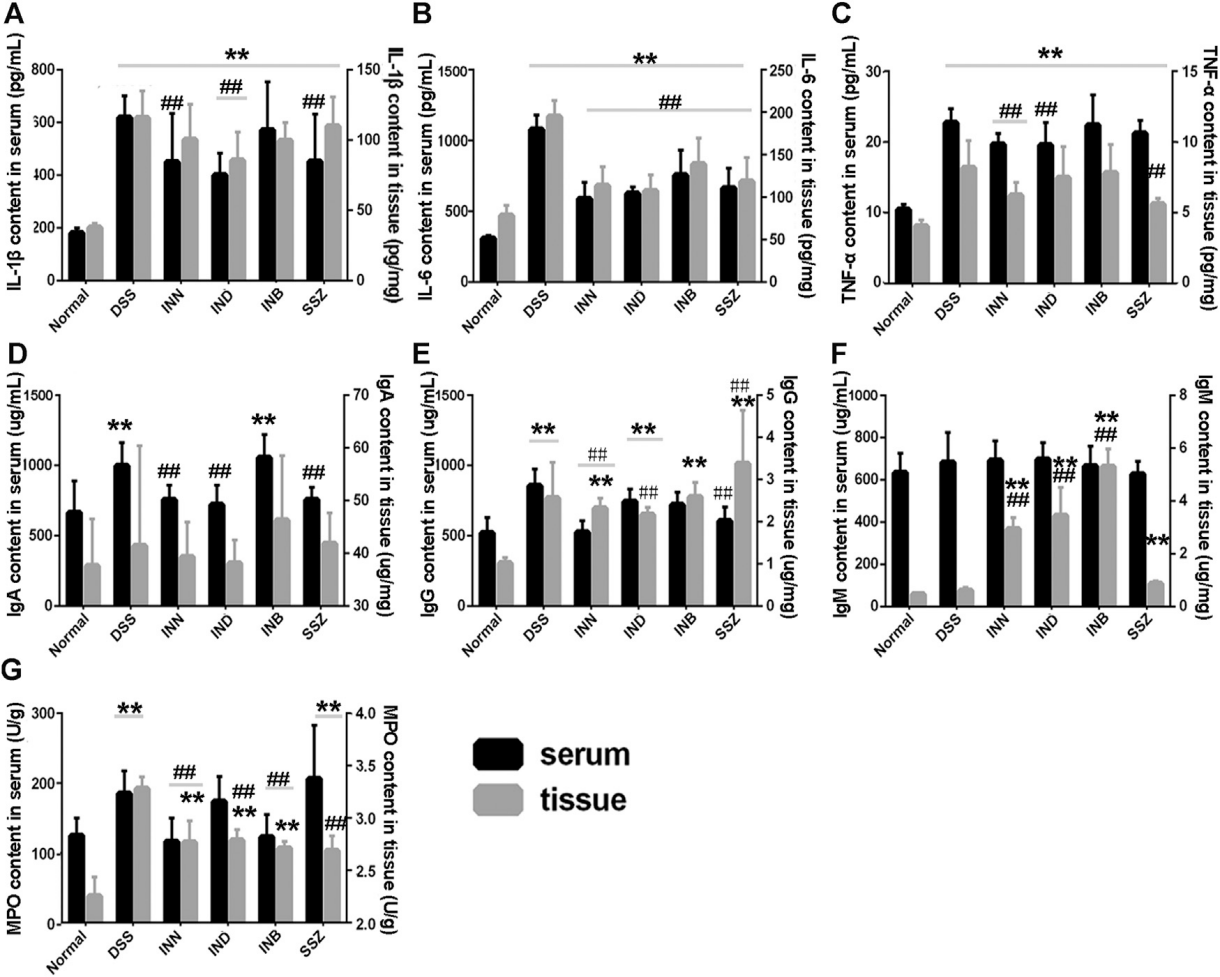
the Changes of IL-1β **(A)**, IL-6 **(B)**, TNF-α **(C)**, and MPO **(D)** both in Serum and tissue. *Vs* normal group, **p < 0.01; *vs* DSS group, ^*▲▲*^ *p* < 0.01. Data are expressed as mean ± SD (*n* = 6).

Levels of cytokine (IL-1β, IL-6, and TNF-α) in serum and tissue were detected by ELISA kits. Levels of IL-1β, IL-6, and TNF-α in serum and tissue were increased significantly in the DSS group (Figures 3A–C). After administration of INN, IND, INB, and SSZ, levels of IL-1β, IL-6, and TNF-α in serum and tissue of mice were decreased in different degrees. The level of IL-1β in serum could be reduced significantly by IND and SSZ and the level of IL-1β in tissue could be reduced significantly by IND and INB. All drugs can significantly reduce the content of IL-6 both in serum and tissue. However, these drugs could not decrease the level of TNF-α.

### The Change of Immunoglobulin Both in Serum and Tissue

Levels of IgA and IgG in serum and tissue were increased significantly in the DSS group (Figures 3D,F). After administration of INN, IND, or SSZ, the level of IgA in serum was decreased significantly and approached normal level. In colon tissue, the level of IgA in all administrative groups had no difference compared with the DSS group. INN also can reduce the IgG level both is serum and tissue compared with the DSS group. The effect of IND and INB is weaker than INN and they just decreased IgG level in serum or tissue. The IgM level of all groups had no difference in serum (Figure 3F). Interestingly, INN, IND, and INB could increase IgM level in tissue compared with normal and DSS groups.

### The Changes of MPO Both in Serum and Tissue

MPO is a functional and activation marker of neutrophils and participates in many inflammatory processes. DSS could significantly increase the level of MPO both in serum and tissue (Figure 3G). INN and INB could reduce the level of MPO to normal in serum. These results suggested that INN, IND, and INB could reduce inflammation in DSS-induced UC mice.

### Expression Levels of TLR4/MyD88/NF-κB Related Proteins in Colon Tissue

**FIGURE 4:**
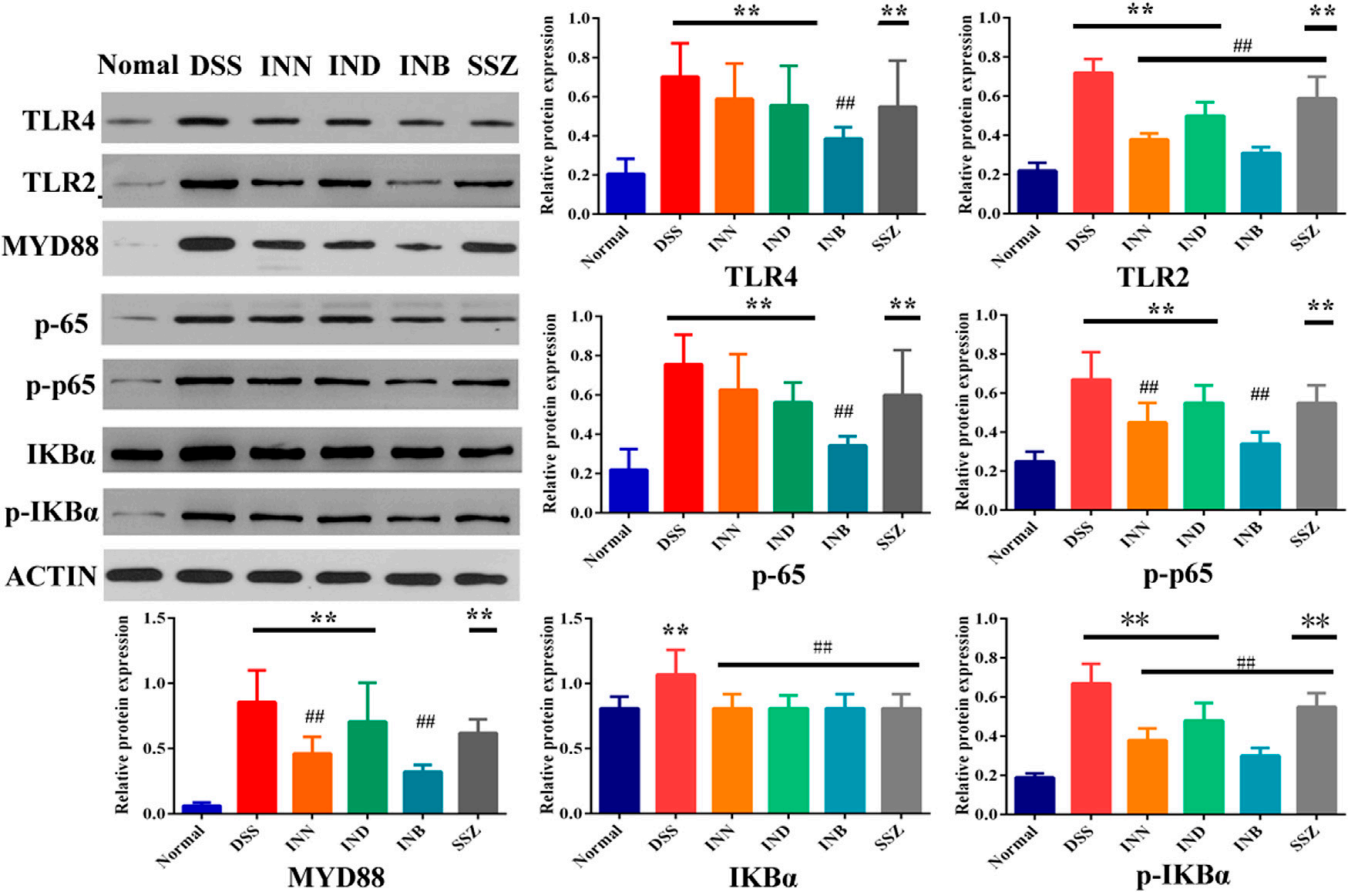
The Expression of proteins in colon tissue. *vs* normal group, **p < 0.01; *Vs* DSS group, ^##^ *p* < 0.01. Data are expressed as mean ± SD (*n* = 3).

To verify whether the therapeutic effect of INN, IND, and INB on UC is related to TRL4/MyD88/NF-κB signaling pathway, expressions of TRL4, TLR2, MyD88, NF-κB p65, p-p65, IKBα, and p-IKBα proteins in colon tissue were detected. After treating with DSS, the expression of TLR4, TLR2, MyD88, p65, p-p65, IKBα, and p-IKBα proteins was significantly increased (Figure 4). INN, IND, INB, and SSZ could decrease expression of these proteins, especially INB. These results showed that INN, IND, and INB can improve UC symptoms through TRL4/MyD88/NF-κB signaling pathway.

### The Effect of INN, IND, and INB on Gut Microbiota

**FIGURE 5:**
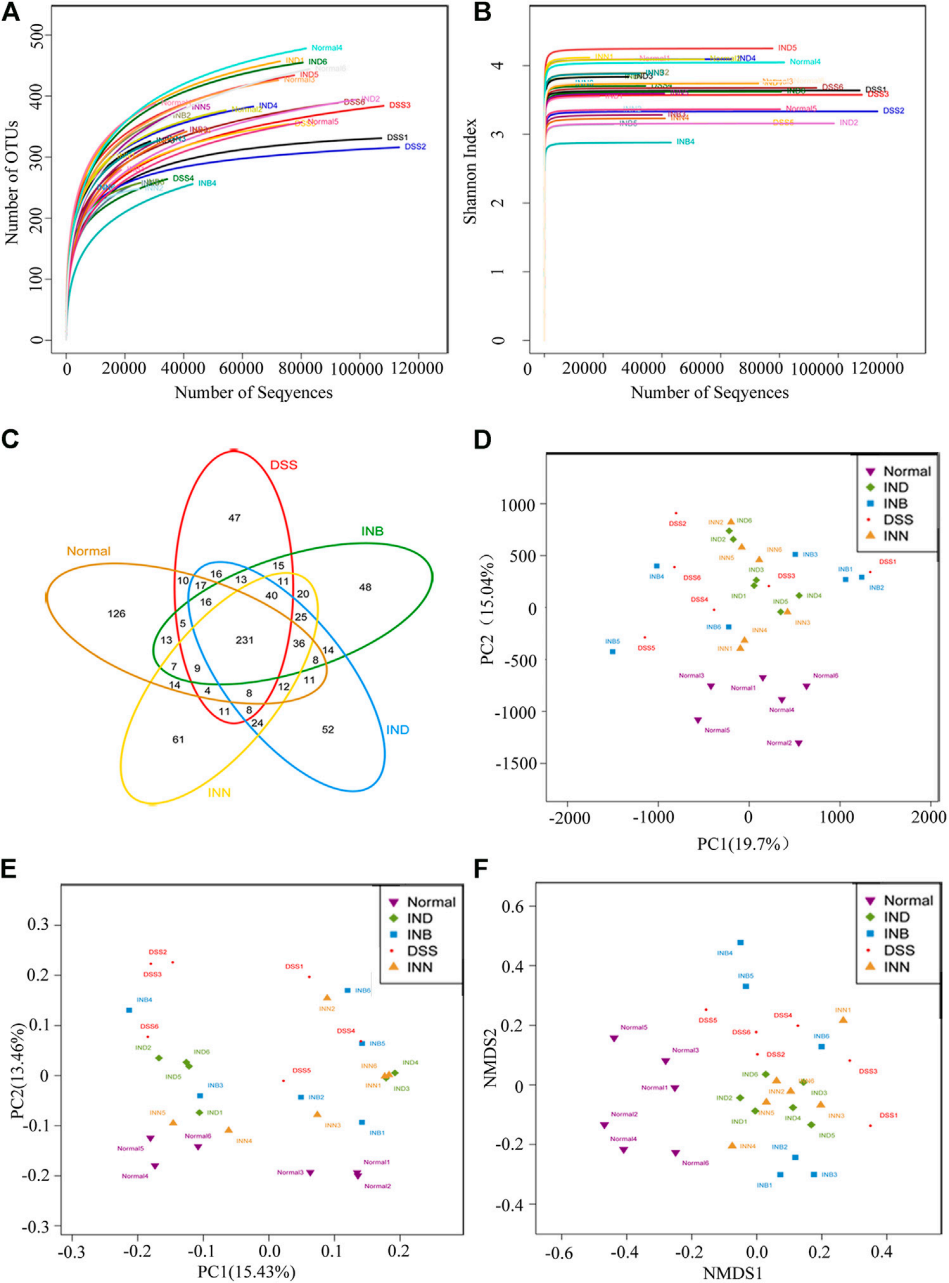
INN, IND, and INB regulation on the disturbed gut microbiota in DSS-induced UC mice **(A)** rarefaction curves determined at the 97% similarity level; **(B)** shannon-Wiener curves of samples; **(C)** venn diagram of OTU in the five groups; **(D)** multiple sample PCA analysis; **(E)** multiple sample PCoA analysis; **(F)** Multiple sample NMDS analysis.

Alpha diversity analysis can reflect abundance and diversity of the microbial community. The rarefaction curve tends to be flat, indicating that the amount of sequencing data is reasonable (Figure 5A). Shannon-Wiener is utilized to reflect the microbial diversity with different sequencing numbers. Its curve trends to flat, indicating the sequencing data is large enough, which can reflect the vast majority of microbial information in samples (Figure 5B). Venn diagram was used to reflect the composition information of species in each group (Figure 5C). These five groups have 231 OTUs overlapped; Normal and DSS groups have 300 OTUs overlapped. DSS and INN groups have 340 OTUs overlapped, DSS and IND groups have 341 OTUs overlapped, and DSS and INB groups have 340 OTUs overlapped. Beta diversity analysis can be used to analyze the diversity of species or function distribution among samples. Results of PCA and PCoA analysis showed that there were significant differences of gut microbiota among the five groups (Figures 5D,E)). NMDS analysis based on β diversity reflects the difference between samples through the distance between points. Samples of normal, DSS, INN, and IND groups did not overlap (Figure 5F), indicating that there were significant differences in intestinal microbial composition between every group. But there were overlaps between DSS and INB groups, suggesting that intestinal microbial composition in the INB group was similar to the DSS group.

### Species Difference Analysis of Gut Microbiota in INN, IND, and INB

**FIGURE 6:**
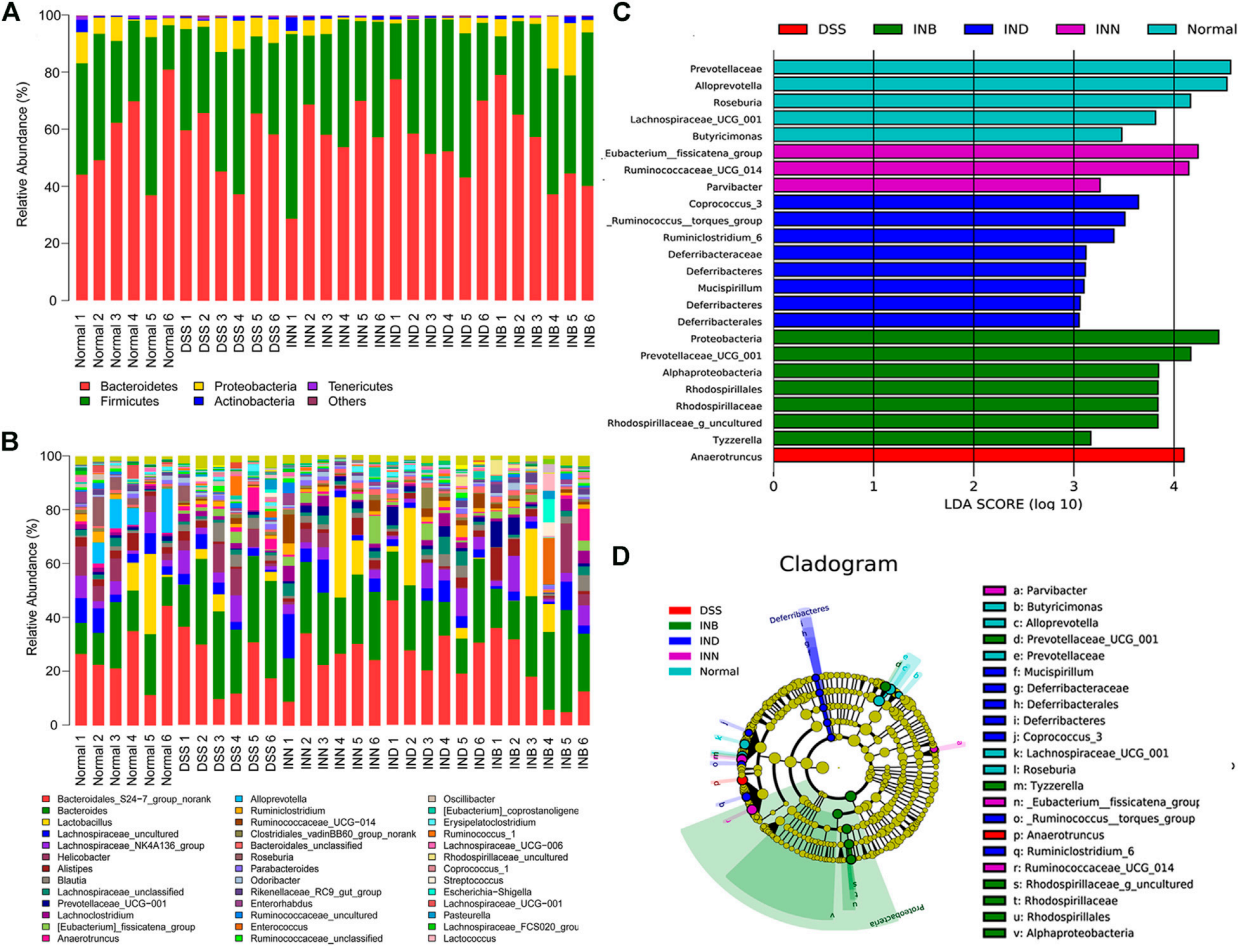
Gut microbial community structures of mice from various groups and difference in dominant microorganisms between groups **(A)** Microbial community bar plot by phylum; **(B)** Microbial community bar plot by genus; **(C)** Distribution histogram based on Lefse; **(D)** Cladogram. (*n* = 6).

Figure 6A showed that *Bacteroidetes*, *Firmicutes*, *Proteobacteria*, *Actinobacteria*, and *Tenericutes* were the predominant species in all groups at the phyla level. Compared with the normal group, *Bacteroidetes* increased significantly and *Firmicutes* decreased significantly in the DSS group. It has been reported that the increase of *Proteobacteria* means the disorder of intestinal flora (Shin et al., 2015). However, the level of *Proteobacteria* in DSS group did not increase, and increased in the INB group in comparison with normal group. The level of *Proteobacteria* decreased in INN and IND groups. Figure 6B showed the difference of intestinal flora in each group at the genus level. *Lactobacillus* are probiotics in the intestine, which can help in digestion and prevent harmful bacteria from adhering to the intestinal epithelium (Azad et al., 2018). The abundance of *Lactobacillus* increased in INN, IND, and INB groups, indicating that these drugs can increase probiotics to protect the intestine. INN and IND could decrease the abundance of *Streptococcus*, most of which are conditional pathogens. *Desulfovibrio*, harmful bacteria, could be returned to normal levels by INN and INB. These results indicated that INN, IND, and INB could regulation the structure of gut microbiota, but the regulation of INB was weakest among them.

LEfSe (LDA effect size) was used to determine the bacterial genera with significant differences among groups. Figures 6C,D showed that the normal group has five special groups, DSS group has one special group, INN group three special groups, IND group has eight special groups, and INB group has seven special groups. An LDA value of more than 3.5 was regarded as the screening standard of dominant microorganisms. The dominant microorganisms in normal group were *Roseburia*, *Alloprevotella*, *Prevotellaceae*, and *Lachnospiraceae_UCG_001*. The DSS group had only one dominant microorganism, namely *Anaerotruncus*. The INN group had two dominant microorganisms: *_Eubacterium__fissicatena_group* and *Butyricimonas*. The IND group had three dominant microorganisms, namely *Coprococcus_3, Ruminiclostridium_6*, and _*Ruminococcus__torques_group*. The INB had the most dominant microorganisms, such as *Rhodospirillaceae*, *Rhodospirillales*, *Alphaproteobacteria*, *Prevotellaceae_UCG_001*, *Proteobacteria,* and *Rhodospirillaceae_g_uncultured*.

## Discussion

TLRs are key factors in the innate immune system. After TLRs are activated, they can transmit inflammatory reaction information through MyD88 dependent signal pathway, mediate the expression and release of inflammatory factors, and produce inflammatory injury (Cheng et al., 2018; Gu et al., 2018). NF-κB is an important inflammatory regulator, which can mediate the transcription and expression of TNF-α, IL-1 β, IL-6, and other inflammatory mediators and growth factors (Kim et al., 2017; Kim et al., 2019; Burgueno and Abreu, 2020). NF-κB-induced inflammatory factors could further sustain and activate NF-κB which aggravates the inflammatory injury. Western blotting results showed that DSS-induced UC could activate TLR4/MyD88/NF-κB signaling pathway and increase the expression of TLR4, MyD88, and NF-κB. But INN, IND, and INB could reduce the expression of TLR4, MyD88, and NF-κB and inhibit activation of TLR4/MyD88/NF-κB signaling pathway, reducing the level of TNF-α, IL-1 β, and IL-6 in serum and tissue.

IND and INB, the main components of INN, can relieve the diarrhea and bloody stool induced by DSS, which is consistent with the previous reports (Gao et al., 2016; Wang et al., 2017). According to the physiological condition of mice, the therapeutic effect of IND and INB is weaker than that of INN, which may be caused by the lower dosage of IND and INB, or other components of INN may also have anti UC effect. In addition, INB has a strong anti-inflammatory effect even at low doses and can effectively inhibit the signal transduction of TLR4, MyD88, and NF-κB p65, indicating that INB may be a promising candidate for UC therapy. In future experiments, the dosage and administration mode of INB could be explored in the treatment of UC.

Immunoglobulin is the classic representative of humoral immunity, which plays an important role in anti-inflammatory and immune regulation (Ma, 2019). IgA is the main immunoglobulin in the intestine, which plays an important role in maintaining the interaction between microorganisms and the intestine in mucosa surface (Pabst, 2012). IgM is the second most abundant homotype in mucosal secretions, which can compensate for IgA deficiency (Liu et al., 2019). IgG is a common antibody in the circulatory system, which is induced by the recognition of "non-self" antigens by systemic immunity, and generally does not produce in the local intestine (Yoshida et al., 2004). A study has shown that the level of immunoglobulin increases with the severity of the disease (Wang et al., 2016). Our experimental results are consistent with this conclusion. However, some studies have found that many patients with IBD have decreased immunoglobulin, which may be related to the progress of the disease and the patient’s own factors (Rai et al., 2015). Therefore, it is necessary to further study the relationship between immunoglobulin and UC.

Intestinal flora in the treatment of UC is a research hotspot at present, as well as a potential new treatment method in the future, which is of great significance to human health. More than 98% of intestinal microorganisms are mainly composed of *Firmicutes*, *Bacteroidetes*, *Actinobacteria,* and *Proteobacteria* (Alam et al., 2020). Studies have shown that the intestinal microflora structure of UC patients will change, mainly manifested by the reduction of flora diversity and *Firmicute* abundance (Walker et al., 2011; Ricciuto et al., 2020). This is basically consistent with our experimental results. The changes of *Proteobacteria* and *Bacteroidetes* abundance are not clear, which may be related to the actual situation of UC patients (Nishida et al., 2018). In this manuscript, the dominant flora in DSS group is *Anaerotruncus*, which is a probiotic. This result may be caused by the body’s stress response. Effects of INN, IND, and INB on the structure of intestinal flora are different. The structure of intestinal flora in INN and IND groups was similar, and the dominant bacteria were *Butyricimonas*, *Eubacterium*, *Coprococcus, Ruminococcus,* and other probiotics. Interestingly, the predominant flora in the INB group was *Proteobacteria*, whose abundance increase meant flora disorder. These results suggest that INB in INN mainly plays an anti-inflammatory role, while IND is mainly used in regulating intestinal flora. Simultaneously, both IND and INB could promote intestinal mucosal repair.

## Conclusion

IND and INB are one of the main anti-UC components of INN. INN could regulate intestinal flora, reduce inflammation, repair intestinal mucosa, and improve the physiological status of DSS-induced UC mice. In addition, inhibition of TLR4/MyD88/NF-κB signal transduction may be one of the most effective mechanisms of INN in the treatment of UC.

## Data Availability

The original contributions presented in the study are included in the article/Supplementary Material, further inquiries can be directed to the corresponding authors.
