# Supplementary figures and images for: Exploring the Mechanism of Indigo Naturalis in the Treatment of Ulcerative Colitis Based on TLR4/MyD88/NF-κB Signaling Pathway and Gut Microbiota
Source: Front Pharmacol. 2021 Jul 22;12:674416. doi: 10.3389/fphar.2021.674416 (PMC8339204; doi:10.3389/fphar.2021.674416)

**MyD88**

**
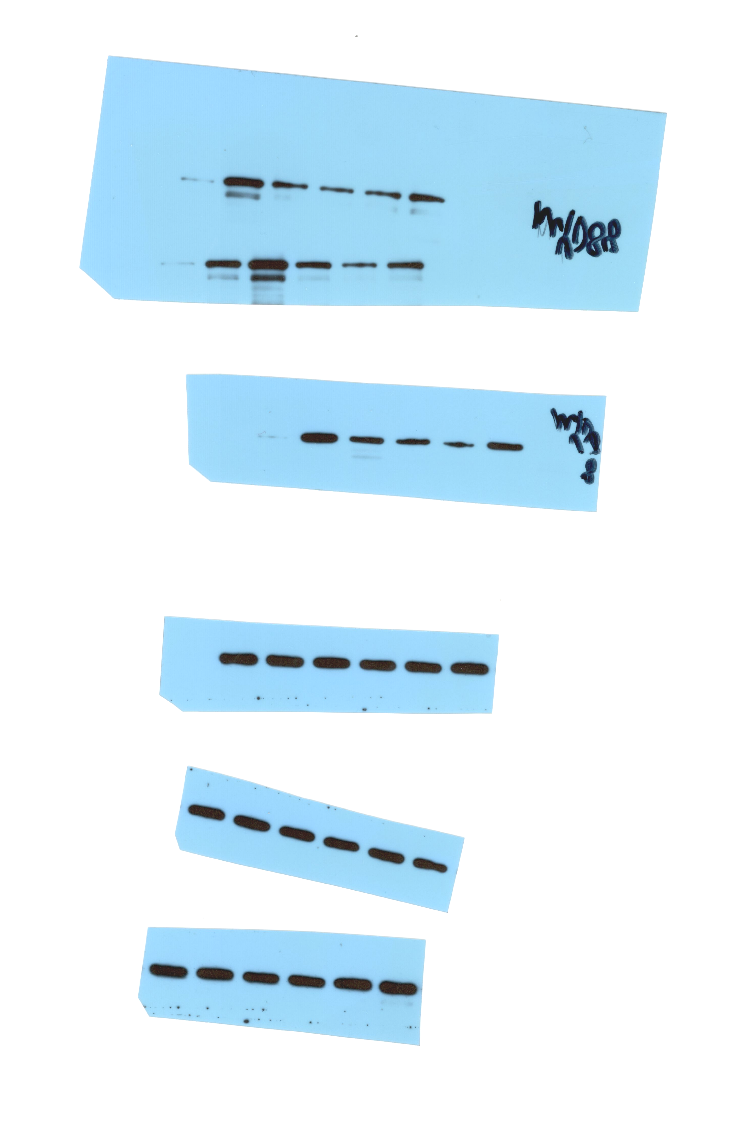
**

**ACTIN**

**
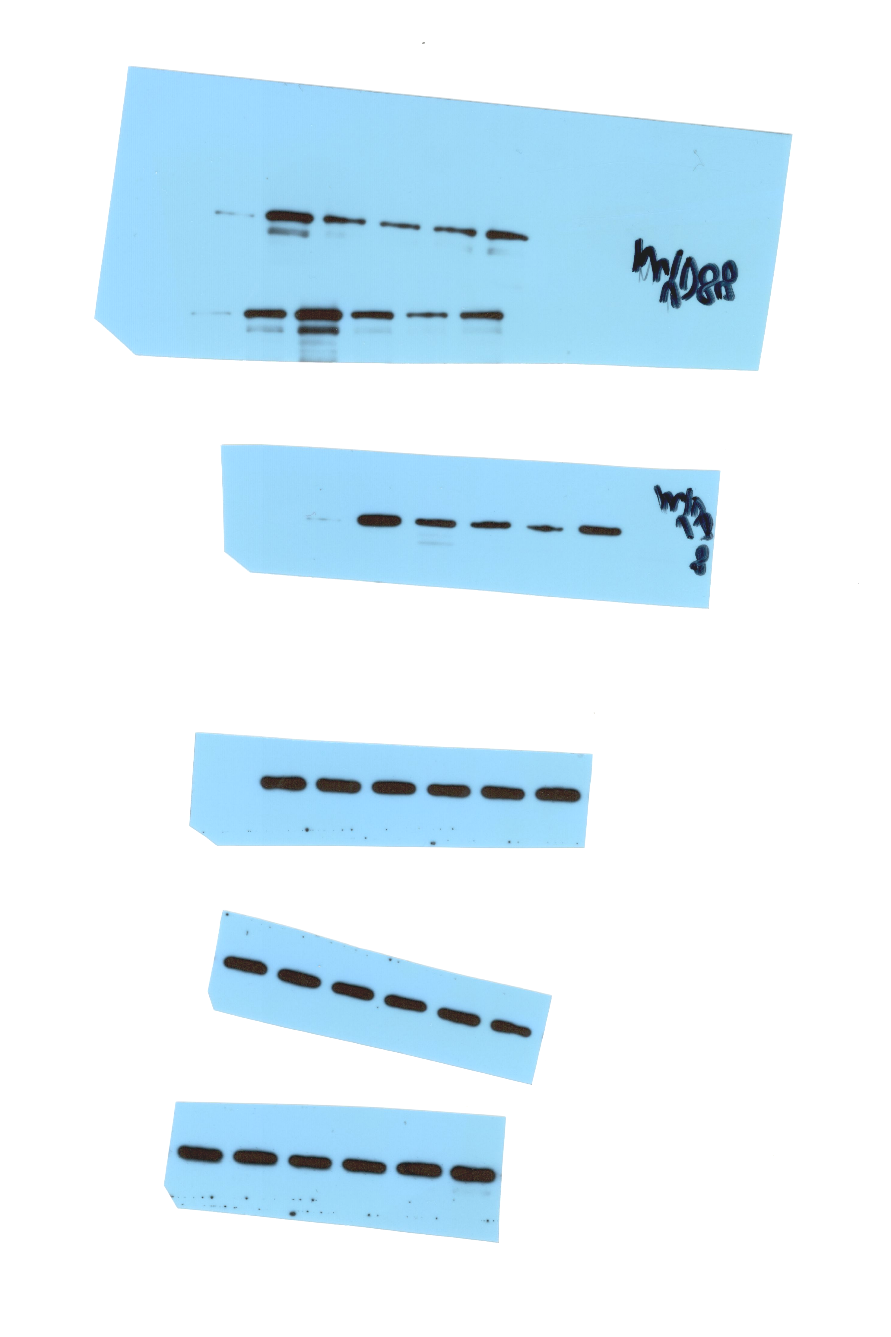
**

**TLR4**

**
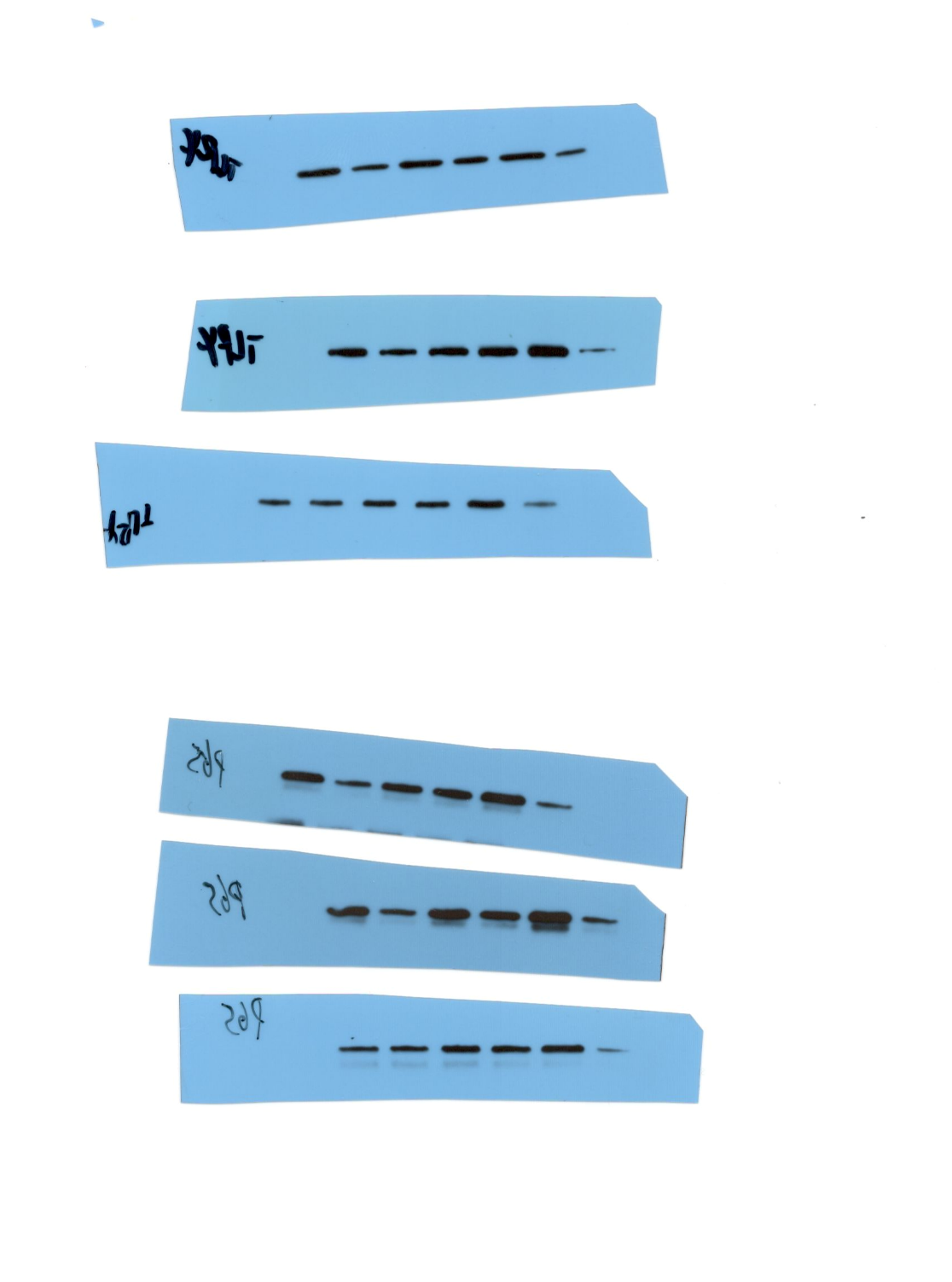
**

**P65**

**
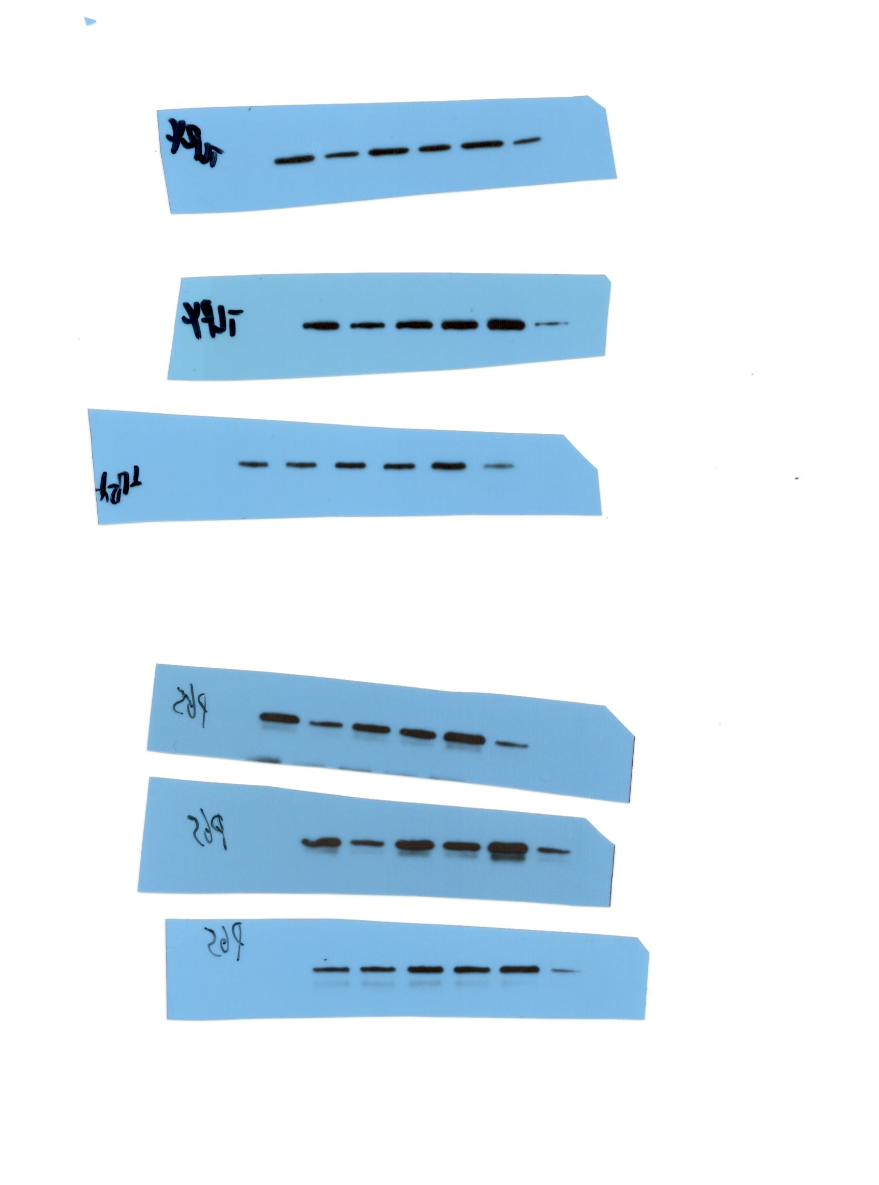
**

**GAPDH**

**
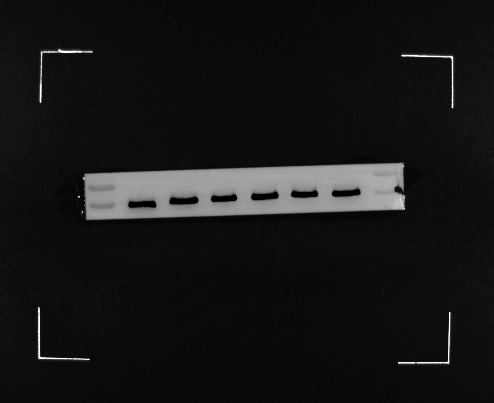

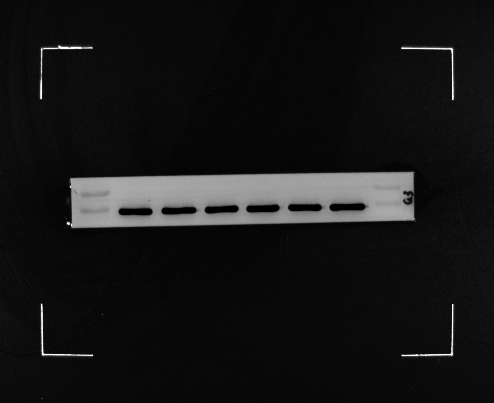

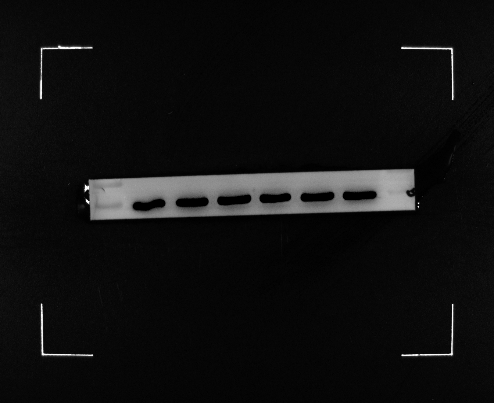
**

**Histone H3**

**
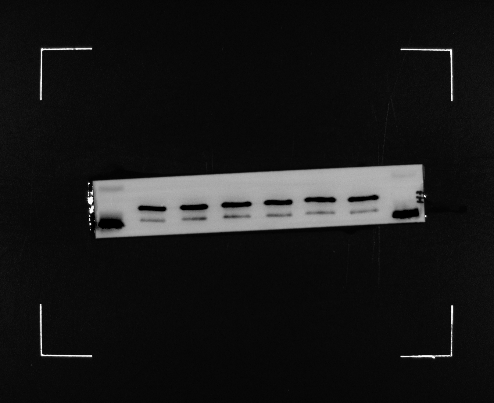

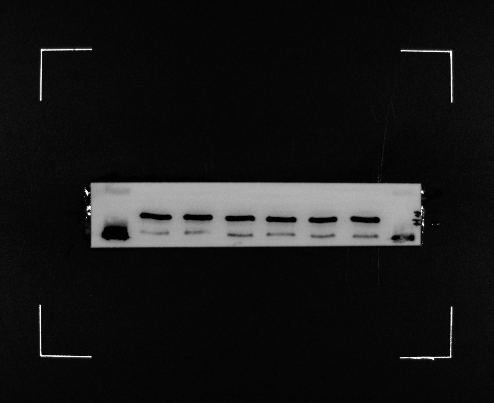

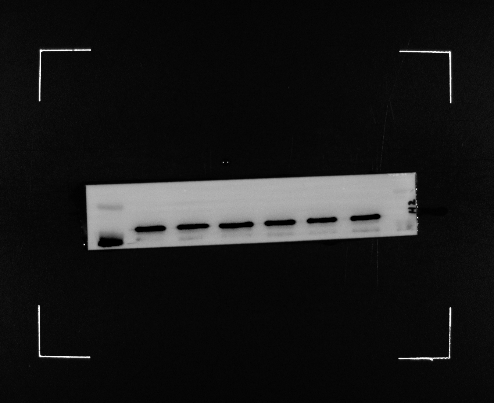
**

**p-p65**

**
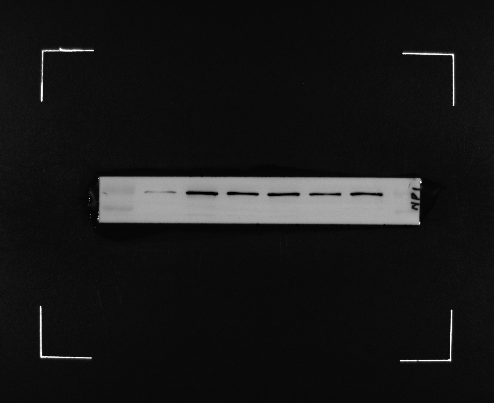

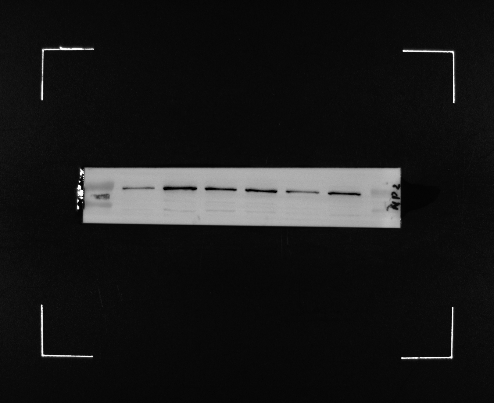
**

**
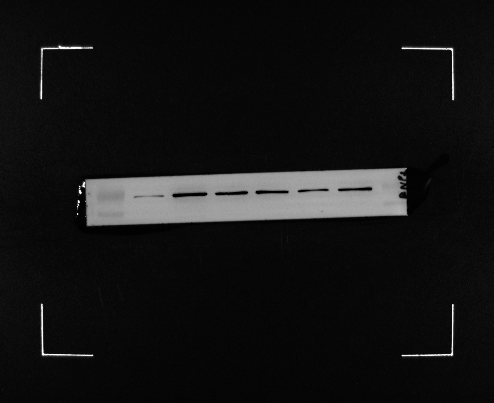
**

**TLR2**

**
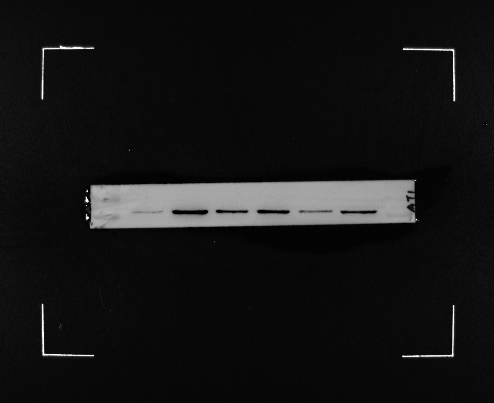

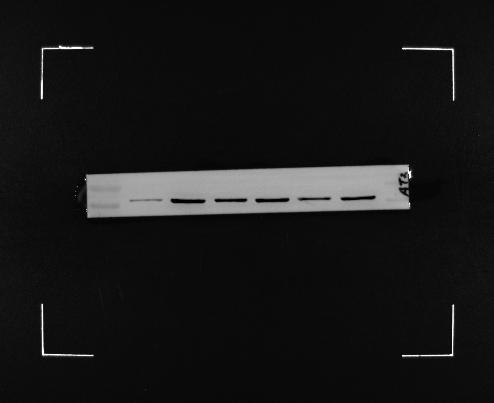

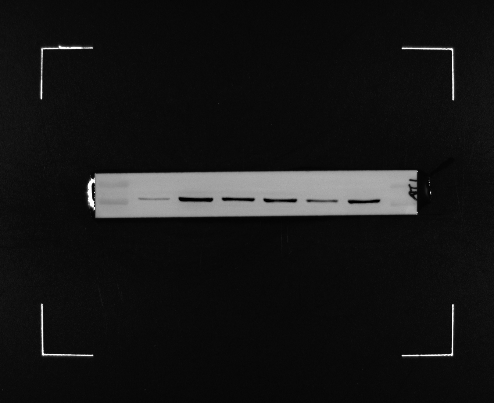
**

**IKBα**

**
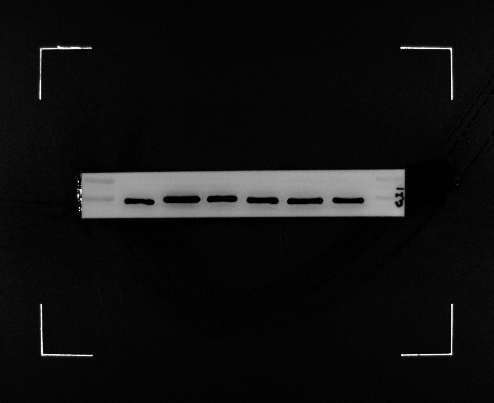

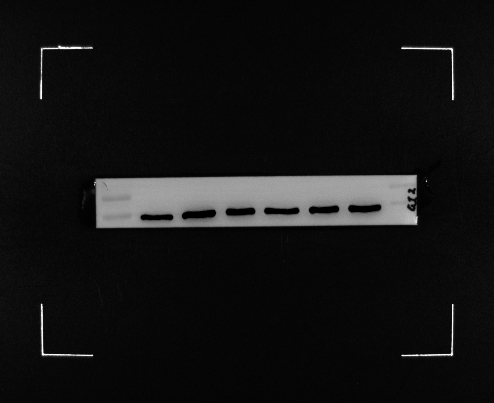
**

**
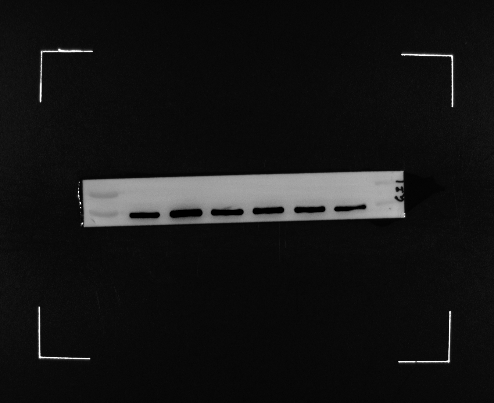
**

**p-IKBα**

**
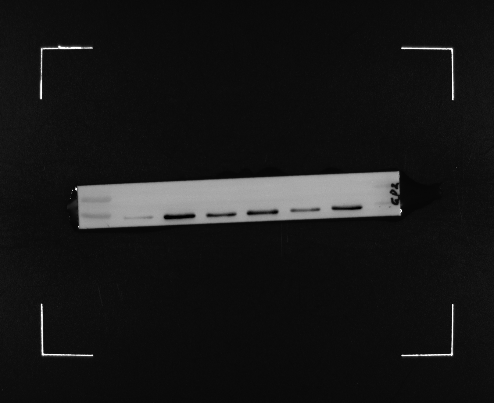

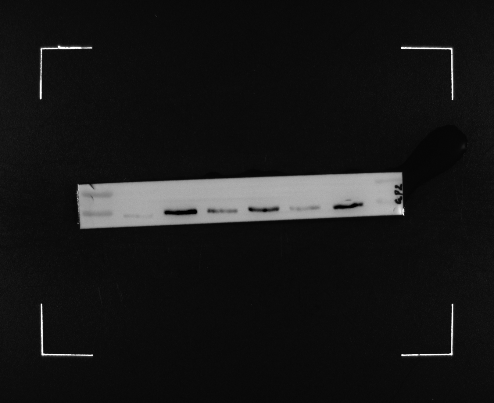

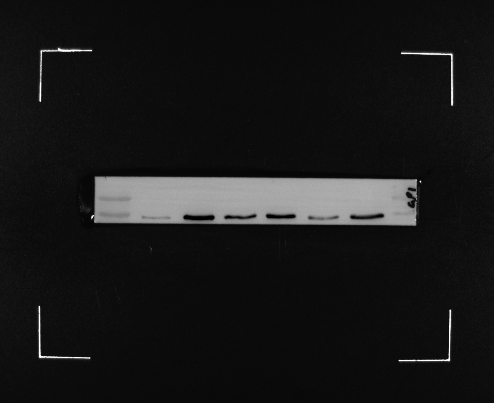
**

Supplement: Supplementary file 1 [file DataSheet2.docx]
