# Supplementary material for: Exploring the Mechanism of Indigo Naturalis in the Treatment of Ulcerative Colitis Based on TLR4/MyD88/NF-κB Signaling Pathway and Gut Microbiota
Source: Front Pharmacol. 2021 Jul 22;12:674416. doi: 10.3389/fphar.2021.674416 (PMC8339204; doi:10.3389/fphar.2021.674416)
Supplement: Supplementary file 2 [file DataSheet1.docx]

**Supplement materials 1**

**Prediction of target of indigo naturalis by molecular docking**

**1 Methods**

The crystal structures of NF-κB, TLR4, MyD88, EPR1/2, JAK2, STAT3 and MAPK were downloaded from RCSB database (<http://www.rcsb.org/>). Download the mol2 format file of indigo and indirubin with 3D structure from PubChem database (<https://www.ncbi.nlm.nih.gov>). PyMOL and ChemDraw 3D software were used to optimize the protein structure and compound structure respectively. Finally, autodock software was used for molecular docking, and PyMOL software was used to visualize the results.

**2. Results**

The lower the binding energy, the stronger the binding force between the compound and the target. The results showed that Indigo and indirubin have strong binding capacity with TLR4, MyD88, NF-κB, JUK, JAK2 and MAPK, especially MyD88 (Figure S1 and Table S1). Therefore, TLR4/MyD88/NF-κB signaling pathway may be the potential mechanism of indigo naturalis.

| **Target** | **Pubmed ID** | **Binding energy of indigo** | **Binding energy of** **indirubin** |
| --- | --- | --- | --- |
| TLR4 | 4R7N | -6.79 | -6.42 |
| MYD88 | 4DOM | -7.91 | -8.49 |
| NF-KB | 2RAM | -6.72 | -7.77 |
| ERK1/2 | 5LCJ | -5.84 | -6.94 |
| JNK | 3PTG | -6.75 | -7.49 |
| MAPK | 6Y4V | -7.71 | -7.83 |
| JAK2 | 3FUP | -7.65 | -7.69 |
| STAT3 | 6NUQ | -5.81 | -5.99 |

**Table S1 Binding affinity of indigo and indirubin to anti-UC related proteins**

**
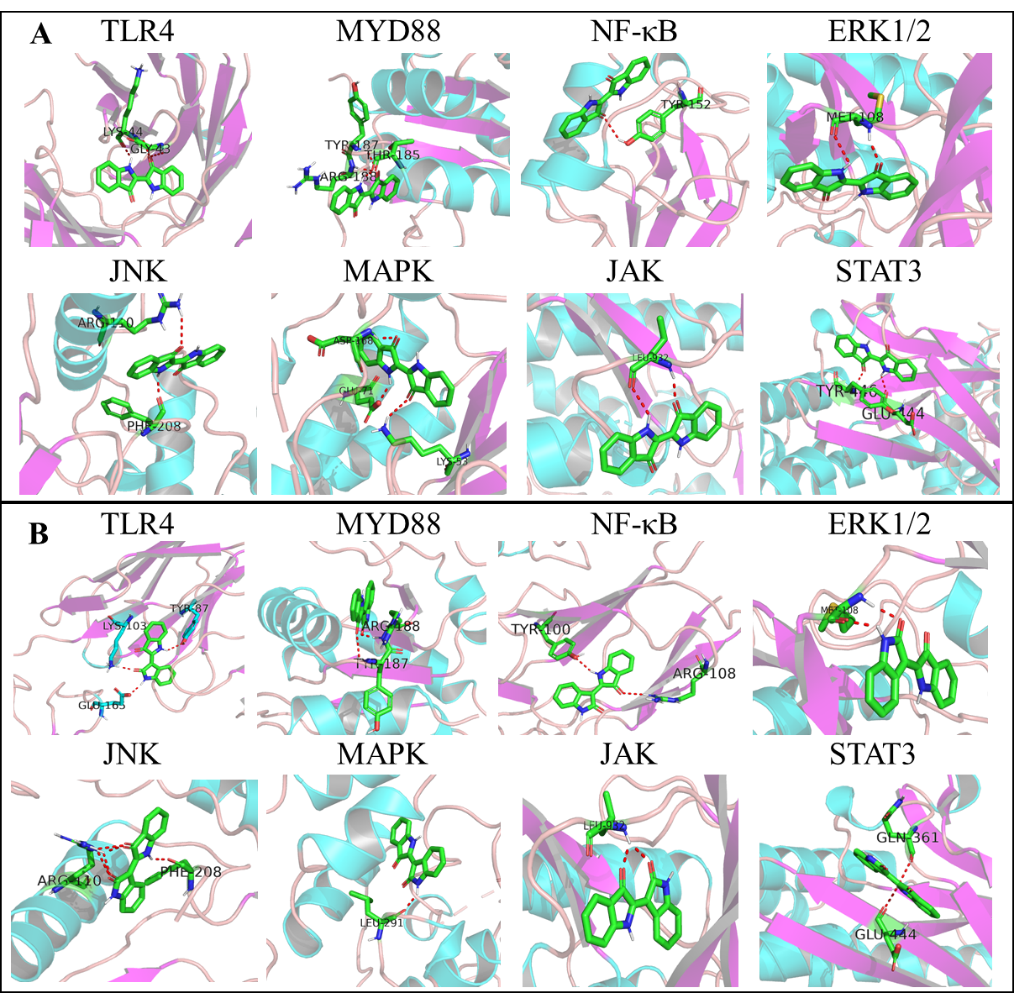
**

**Figure S1 Optimal docking conformations of target proteins with indigo (A) and indirubin (B)**

**Supplement materials 2**

**Therapeutic effect of** **indigo naturalis with different doses on ulcerative colitis**

**1 Method**

**1.1 Animals**

Male BalB/c mice (20 ± 2 g) were purchased from Chengdu Dashuo Laboratory Animal Co., Ltd (Permit No. SCXK (chuan) 2015-30, Chengdu, China). The animals were kept under controlled conditions of temperature 20 ± 0.5˚C, humidity 55 ± 5% and a 12 h light / dark cycle.

**1.2 Therapeutic Potential of indigo naturalis with different doses on a DSS-induced UC mouse model**

The mice were randomly divided into six groups: Normal group, DSS group, INN-L group, INN-M group, and INN-H group (n=6). After one week of adaptive feeding, the mice in DSS, INN-L group, INN-M group, and INN-H group were given 3% DSS drinking water (w/v) to establish UC model and the mice in normal group were given distilled water. On the fourth day after drinking DSS solution, the mice in INN, IND, INB and SZZ groups were given intragastric administration of INN-L (100 mg/kg), INN-M (200 mg/kg), and INN-H (400 mg/kg) respectively. All mice received DSS drinking water for 7 days and drug therapy for 7 days. The weight and feces of mice was monitored and recorded every day throughout the experiment. What’s more, the health status of mice was evaluated according to the disease activity index (DAI), which is normal stool (0), soft stools (1), soft stools and slight bleeding (2), loose stools and slight bleeding (3), gross bleeding (4).

**2 Results**

The weight reflects the health of the mice. The results showed that After drinking 3% DSS 4, the weight of mice decreased significantly (Figure S2A). The intervention of indigo naturalis could reverse the weight loss of UC mice. DAI score showed that the DAI score of DSS group was significantly increased (Figure S2B). The intervention of indigo naturalis can slow down the rise of DAI score, even reduce DAI score, which indicates that indigo naturalis can effectively improve the symptoms of hematochezia and diarrhea in UC mice. In addition, the colonic length of UC mice induced by DSS was significantly shorter, and the colonic length could be restored after intervention of indigo naturalis (Figure S2C). According to the weight change, DAI score and colon length, it was found that there was no significant difference in the efficacy of different doses of indigo naturalis. Comprehensive consideration, 200 mg / kg dose was selected for follow-up study.


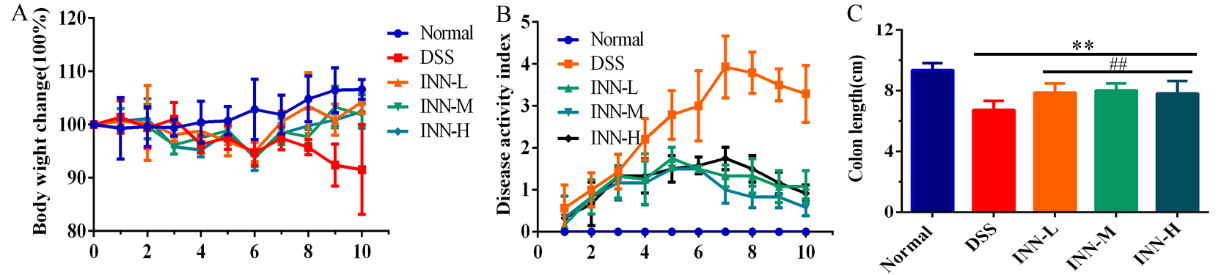


**Figure S2 Effects of INN, IND, INB and SZZ treatment on the of DSS-induced UC mice after 7 days of continuous gavage.**

**(A) body weight change in various groups; (B) disease activity index in various groups; (C) colon length in various groups; Vs normal group, **P < 0.01; Vs DSS group, ##P<0.01. Data are expressed as mean ± SD (n = 6).**
